# Discovery of the male of a rare aulacid wasp, *Pristaulacus emarginaticeps* Turner, 1922 (Hymenoptera: Aulacidae) from Vietnam and Laos

**DOI:** 10.3897/BDJ.6.e26198

**Published:** 2018-05-17

**Authors:** Keita Kuroda

**Affiliations:** 1 Faculty of Agriculture, Ehime University, Matsuyama, Japan

**Keywords:** Evanioidea, description of male, new distribution record

## Abstract

**Background:**

*Pristaulacus emarginaticeps* Turner, 1922 was described from a single female from Hòa Bình Province, Vietnam and it has remained the only recorded specimen.

**New information:**

The male of *Pristaulacus emarginaticeps* Turner, 1922 is described for the first time and the species is newly recorded from Hà Tĩnh Province, Vietnam and Houaphanh Province, Laos.

## Introduction

The *Pristaulacus comptipennis* species group is characterized by the deep occipital emargination of the head (most obvious in dorsal view as in Fig. 2c). This species group was first defined in the phylogenetic analyses by Turrisi et al. (2009) and subsequently formally defined and revised by Turrisi and Smith (2011). It is endemic to South east Asia and four species, *Pristaulacus emarginaticeps* Turner, 1922, *P. excisus* Turner, 1922, *P. lagrecai* Turrisi and Smith, 2011 and *P. vietnamensis* Turrisi and Smith, 2011, have so far been recorded from Vietnam. Amongst them, *P. emarginaticeps* was described from a single female (Turner 1922) and no additional specimens have been recorded (Turrisi and Smith 2011). In samples collected from Vietnam and Laos, I found a female and two males of this species. In this paper, the male morphology of *P. emarginaticeps* is described for the first time and the species is newly recorded from Laos.

## Materials and methods

Examined specimens were purchased from a native collector. They were dried and deposited in Ehime University Museum, Matsuyama, Japan (EUMJ). Male genitalia were treated with 10% potassium hydroxide at 60°C for approximately one hour. The genitalia were rinsed with 70% ethanol and mounted in glycerine for observation and illustration. The dissected genitalia were then stored in microvials with glycerine and pinned with the specimen. Morphological observations were made under a Nikon SMZ1500 stereomicroscope. Genitalia illustrations were drawn under a Leica M205C stereomicroscope. All photographs were taken by a Nikon Digital Sight DS-Fi1 camera attached to a Leica S8APO stereomicroscope. Several partially focused images were combined and post-processed using Adobe Photoshop® CS6. Terminology follows Huber and Sharkey (1993) except the surface sculpture, which follows Eady (1968). A distributional map was made using SimpleMappr (Shorthouse 2010).

## Taxon treatments

### Pristaulacus emarginaticeps

Turner, 1922

Pristaulacus emarginaticeps Turner, 1922: 270.

#### Materials

- **Type status:** Other material. **Occurrence:** recordedBy: native collector; individualCount: 2; sex: 2 males; lifeStage: adult; **Taxon:** scientificName: Pristaulacus emarginaticeps; order: Hymenoptera; family: Aulacidae; **Location:** country: Vietnam; countryCode: VN; stateProvince: Hà Tĩnh Province; **Identification:** identifiedBy: Keita Kuroda; dateIdentified: 2018; **Event:** eventDate: May 2017; **Record Level:** language: English; institutionCode: EUMJ; basisOfRecord: PreservedSpecimen
- **Type status:** Other material. **Occurrence:** recordedBy: J. Yamasako; individualCount: 1; sex: 1 female; lifeStage: adult; **Taxon:** scientificName: Pristaulacus emarginaticeps; order: Hymenoptera; family: Aulacidae; **Location:** country: Laos; countryCode: LAO; stateProvince: Houaphanh Province; locality: Mt. Phou Pan, Ban Saleui, near Xam Neua; **Identification:** identifiedBy: Keita Kuroda; dateIdentified: 2018; **Event:** eventDate: 5 May 2003; **Record Level:** language: English; institutionCode: EUMJ; basisOfRecord: PreservedSpecimen

#### Description

**Figure 1.**
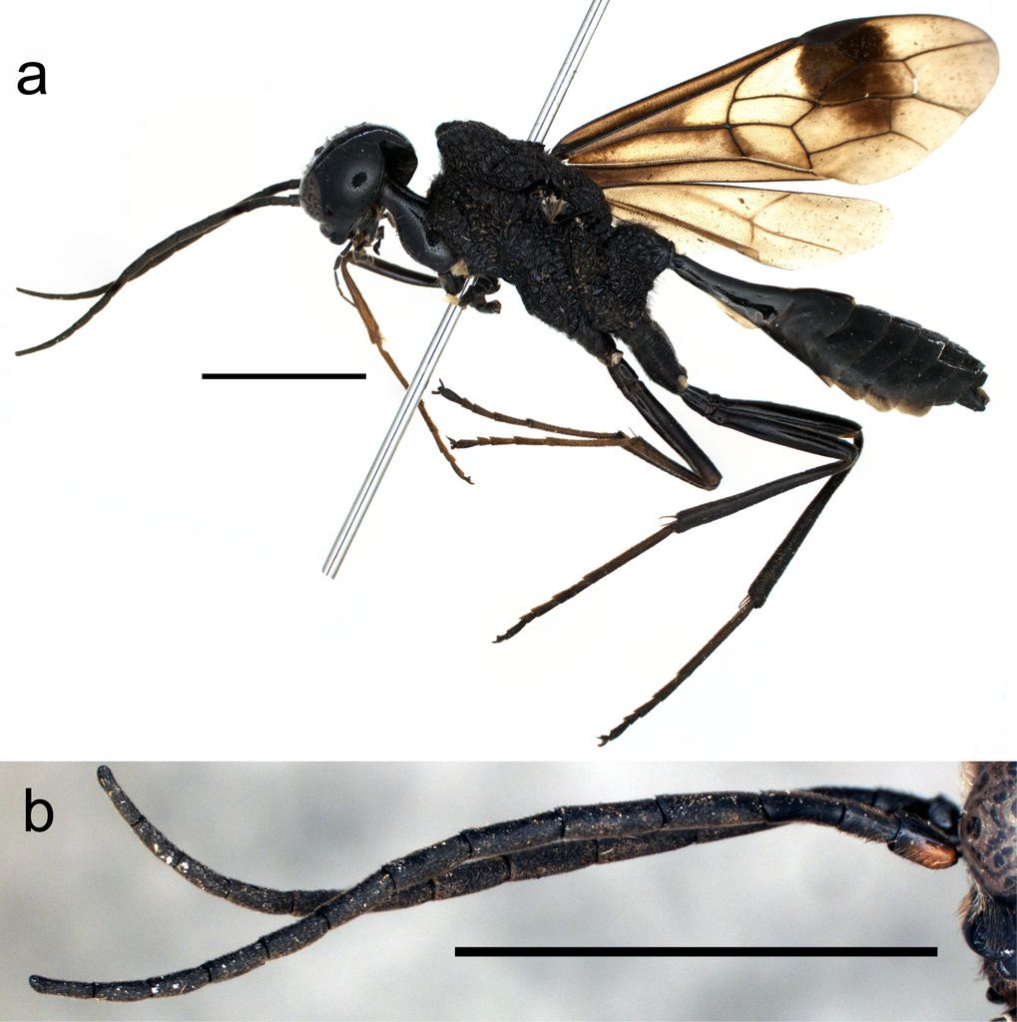
*Pristaulacus emarginaticeps* male. Scale bars 4.0 mm. **a**: lateral habitus; **b**: antennae.

Description of male (Fig. 1a). Colour black; scape entirely reddish-brown; segments 1 and 2 of maxillary palpi brown; apical and basal 1/4 of fore femur brown; fore tibia and tarsus brown; apical and basal tips of mid femur brown; mid tibia and tarsus brown to black; apical and basal tips of hind femur brown; hind tibia brown to black; spurs brown; wings hyaline and tinged with brown, stigma black and vein black to brown; apical half of fore wing strongly infuscate, area along veins *M*+*Cu*, 1*cu-a* and basal half of *A* infuscate; setae brown to light brown except setae on mandible and clypeus light reddish-brown, setae on maxillo-labial palpus and paraocular area pale yellow.

**Figure 2.**
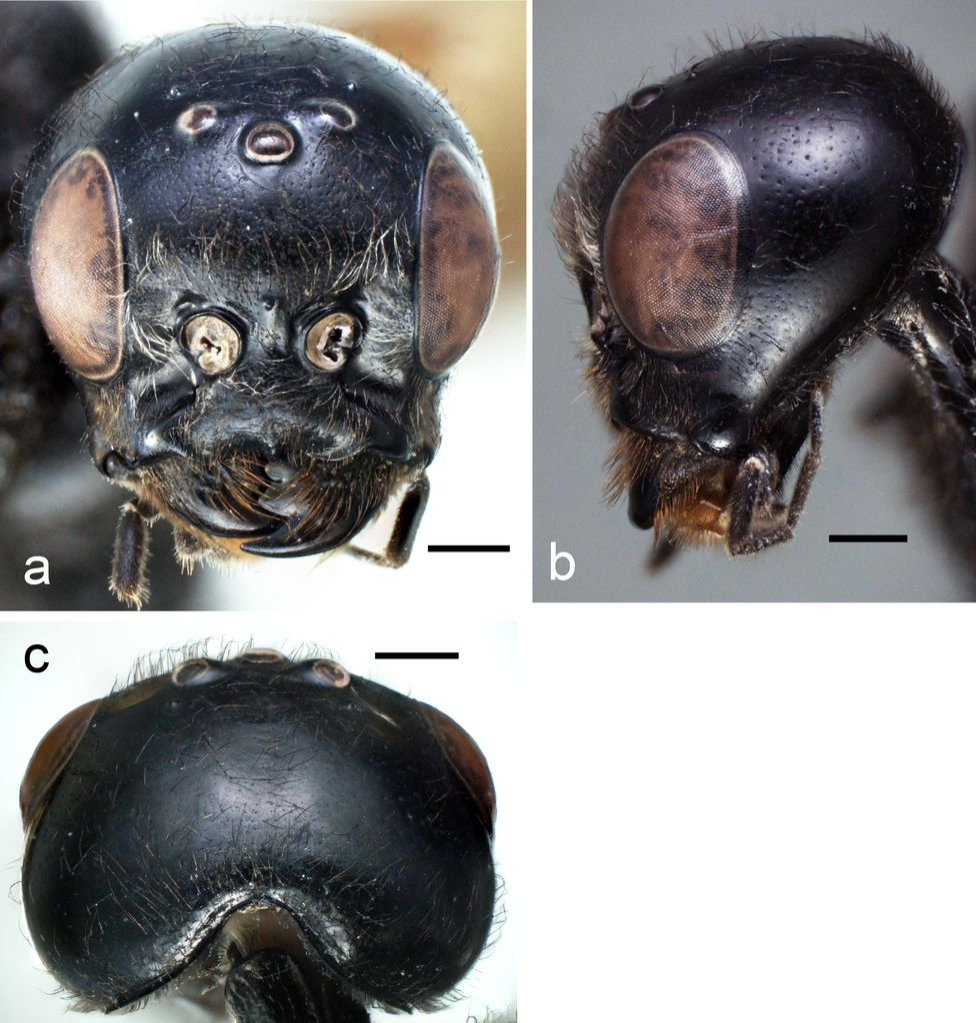
*Pristaulacus emarginaticeps* male. Scale bars 0.5 mm. **a**: head, frontal view; **b**: head, lateral view; **c**: head, dorsal view.

Head (Fig. 2a-c) smooth and shiny, 0.6–0.7 times as long as wide; malar space 0.3 times as long as eye height; median portion of occipital margin strongly grooved; lower 2/3 of occiput with long setae with punctures; occipital carina distinct and complete; vertex, temple and frons sparsely punctate with setae; POL/OOL = 1.2–1.3; antennal socket situated at lower level of eye; face punctuate with long setae; paraocular area with long setae and punctures; clypeus punctate and with dense setae; mandible smooth, basal 2/3 densely punctate with evenly distributed strong setae (Fig. 7a); antenna (Fig. 1b) with 11 flagellomeres, finely punctate with setae and 5.0 times as long as head length; scape 1.6–1.9 times as long as wide; pedicel 2.0–2.2 times as long as wide; 1st flagellomere 2.2–2.6 times as long as wide, 0.7 times as long as 2nd.

**Figure 3.**
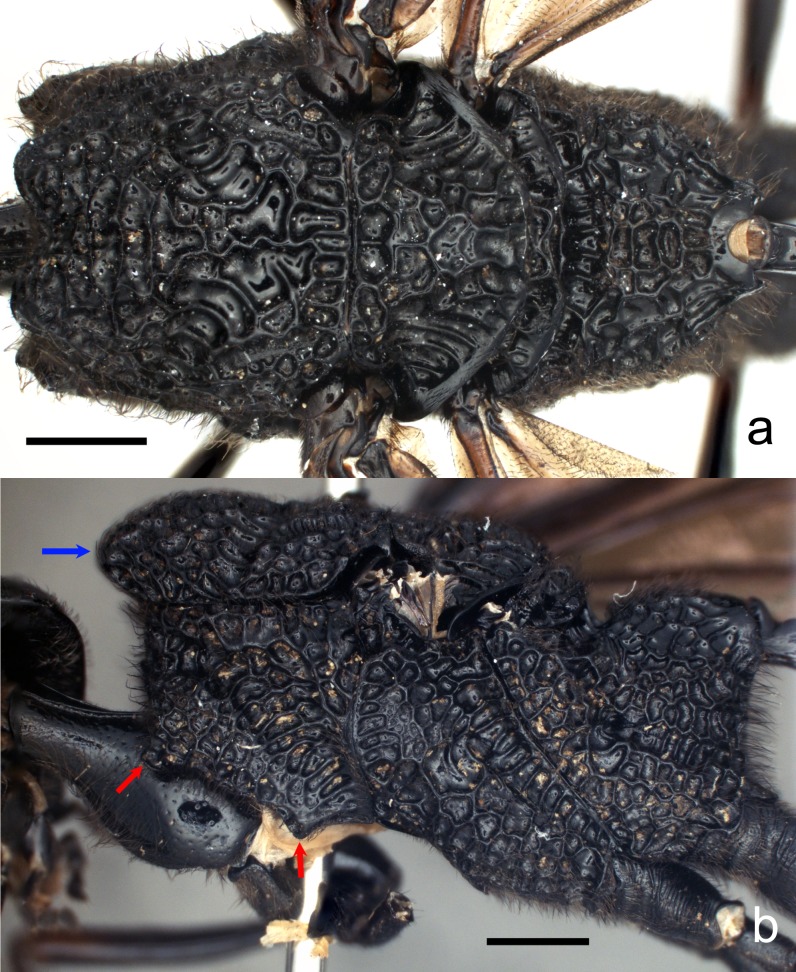
*Pristaulacus emarginaticeps* male. Scale bars 1.0 mm. **a**: mesosoma, dorsal view; **b**: mesosoma, lateral view. (Blue arrow shows the mesoscutal prominence and red arrows show tooth-like processes.)

Mesosoma (Fig. 3a-b) shiny, punctate with fine setae; pronotum reticulate rugose to foveolate, with diagonally transverse canaliculate groove and with 2 tooth-like processes on antero-ventral margin (red arrows in Fig. 3b), one antero-ventral other ventral; mesoscutum reticulate rugose with a pair of lobes on anterior margin projecting strongly forward, the front profile of mesoscutum prominent and slightly pointed in lateral view (blue arrow in Fig. 3b); notauli canaliculate, deep and wide, meeting at median portion of mesoscutum; mesoscutellum reticulate rugose; axillula reticulate rugose to canaliculate; mesopleuron reticulate rugose; metanotum reticulate rugose; metapleuron reticulate rugose; propodeum reticulate rugose except anterior marginal groove longitudinally canaliculate.

Legs: Coxae setose; fore coxa smooth; mid and hind coxae trans-striate on dorsal side; tibiae and tarsi with dense setae and punctures; hind basitarsus 9.2–11.0 times as long as wide; tarsal claws with 6 tooth-like processes.

**Figure 4.**
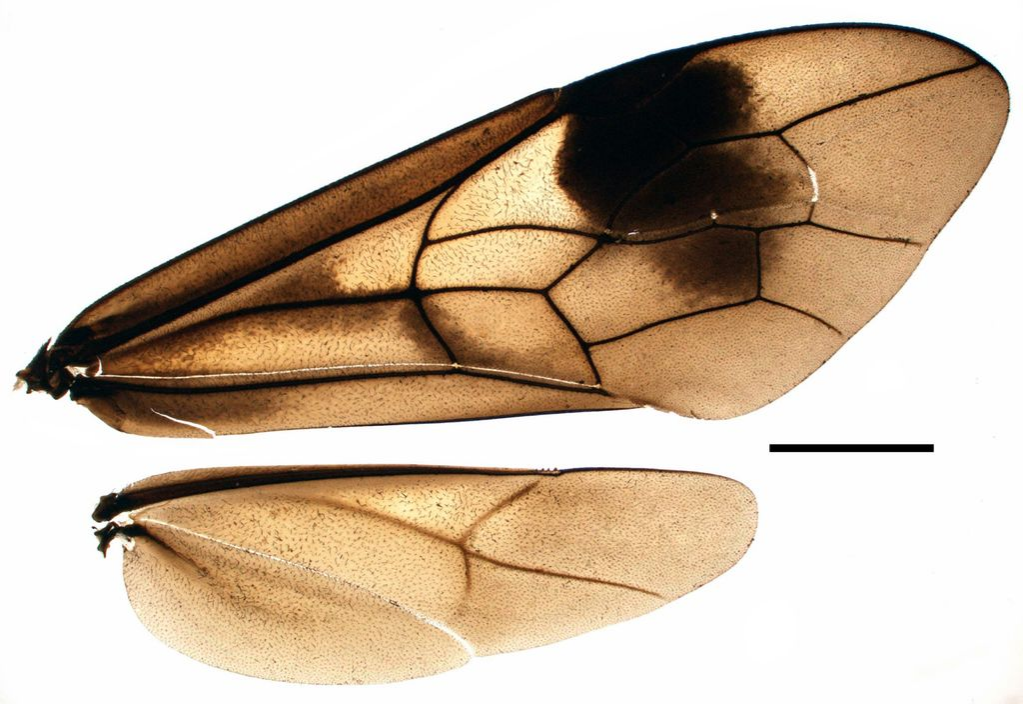
*Pristaulacus emarginaticeps* male wings. Scale bar 2.0 mm.

Wings (Fig. 4): Fore wing 2.8–3.0 times as long as wide, apex rounded; hind wing weakly tapering toward rounded apex, 3.6–3.9 times as long as wide and with 4 distal hamuli.

Metasoma pyriform in lateral view, shiny and covered with sparse setae and punctate; petiole 2.9–3.2 times as long as wide.

**Figure 5.**
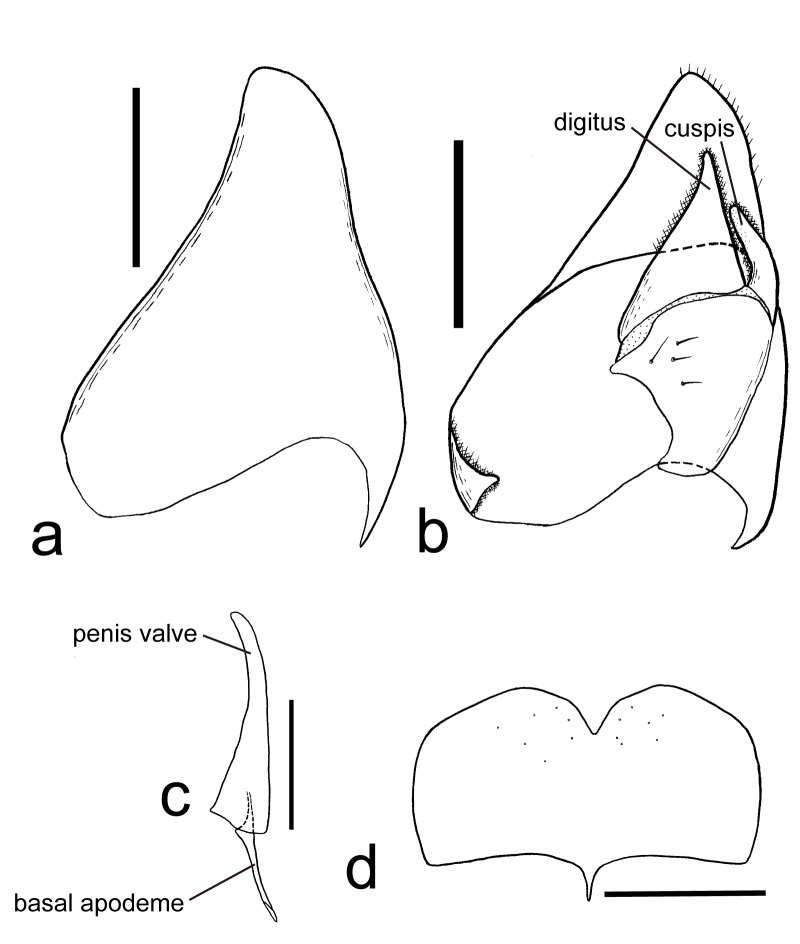
*Pristaulacus emarginaticeps* male genitalia. Scale bars 0.5 mm. **a**: lateral view of left paramere, setae omitted; **b**: mesal view of right paramere; **c**: aedeagus, lateral view; **d**: subgenital plate. (Terminologies of genitalia are shown in black letters, respectively.)

Genitalia: Apical margin of subgenital plate emarginate medially (Fig. 5d); paramere subtriangular (Fig. 5a-b); cuspis finger-like shape, moderately curved and its apex punctate (Fig. 5b); digitus triangular (Fig. 5b); penis valve slightly curved ventrally (Fig. 5c); basal apodeme of aedeagus weakly curved dorsally.

Measurements. Body length 18.5–20.0 mm. Length of fore wing 13.6–14.1 mm.

##### Measurement of female from Laos

Body length 16.3 mm. Length of fore wing 13.6 mm. Ovipositor 13.2 mm. Head 0.8 times as long as wide; malar space 0.3 times as long as eye height; POL/OOL = 1.4; antenna 3.6 times as long as head length; scape 1.6 times as long as wide; pedicel 2.2 times as long as wide; 1st flagellomere 2.5 times as long as wide, 1.5 times as long as 2nd; hind basitarsus 7.9 times as long as wide; fore wing 3.0 times as long as wide, apex rounded; hind wing 3.9 times as long as wide; Petiole could not be measured because it was deformed; ovipositor 1.0 times as long as fore wing.

#### Diagnosis

Male and female. Pronotum with 2 tooth-like marginal processes laterally, one anteroventral other ventral (Fig. 3b); mesoscutum with a pair of strongly projecting forward lobes on anterior margin (Fig. 3a), the front profile of mesoscutum prominent and slightly pointed in lateral view (Fig. 3b); notauli canaliculate, deep and wide (Fig. 3a); tarsal claws with 6 tooth-like processes.

#### Distribution

Laos (1 female from Houaphanh Province), Vietnam (holotype female from Hòa Bình Province, 2 males from Hà Tĩnh Province).

#### Biology

Examined specimens were collected in May and the holotype was collected in August.

## Discussion

**Figure 6.**
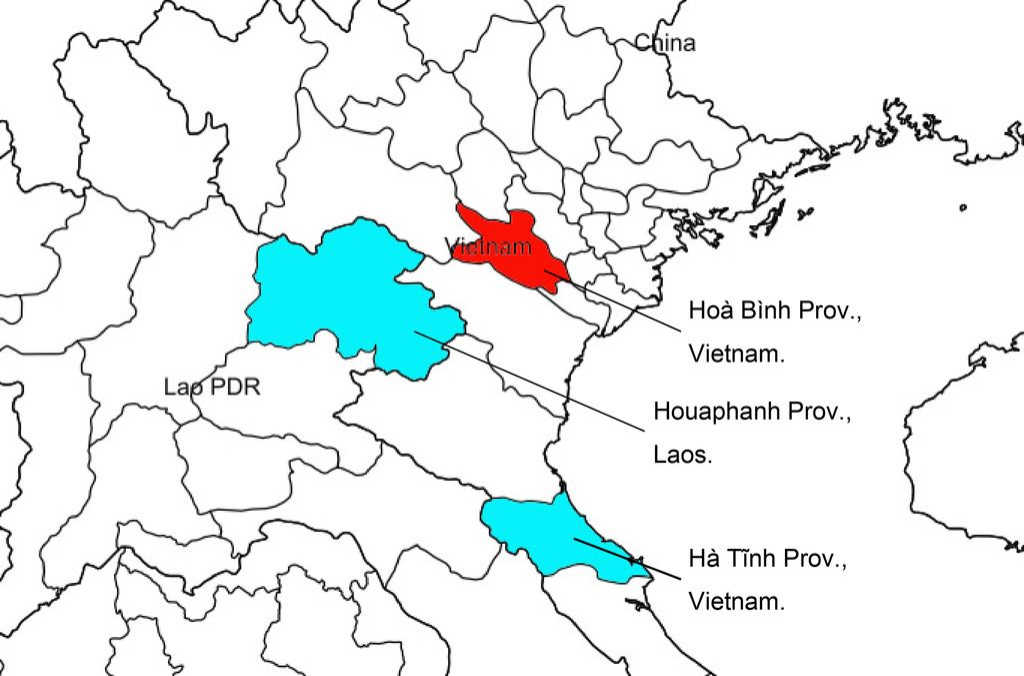
Distribution map of *Pristaulacus emarginaticeps*, new localities (blue) and type locality (red).

**Figure 7.**
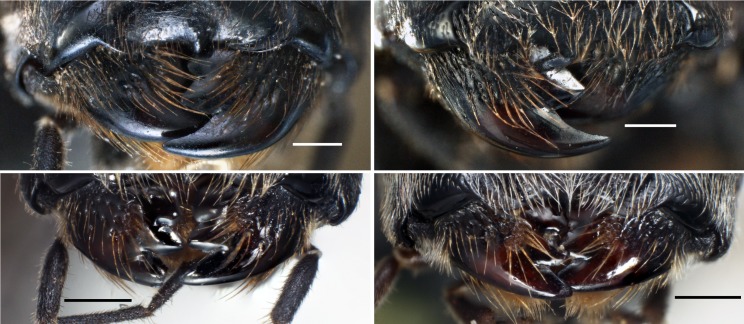
*Pristaulacus* spp. outer faces of mandibles (Figs. 7c-d from Konishi and Kikuchi (2016) on page 26). Scale bars 0.3 mm. **a**: *P. emarginaticeps* male; **b**: *P. emarginaticeps* female; **c**: *P. comptipennis* female; **d**: *P. insularis* female.

The species is newly recorded from Laos and an additional locality is added from Vietnam (Fig. 6). According to Konishi and Kikuchi (2016), the surface sculpture and the distribution pattern of setae on the outer face of the mandible can be used as diagnostic characters in the genus *Pristaulacus*. In *P. emarginaticeps*, the outer face of the mandible is smooth, without a patch of setae or dense punctures and with evenly distributed strong setae present on its basal 2/3. The outer face of the mandible is also smooth in *P. comptipennis* (Fig. 7c) and *P. insularis* (Fig. 7d), but they show different distribution patterns of the setae and punctures (Fig. 7a-b), indicating that the distribution pattern of setae and punctures is a diagnostic character of *P. emarginaticeps*.

Amongst the 27 species so far known in the *P. comptipennis* species group (Turrisi and Smith 2011, Turrisi and Madl 2013, Turrisi 2014, Chen et al. 2016, Turrisi and Nobile 2016), the male has been reported only for four species and male genitalia have been illustrated in three species (i.e. *P. boninensis, P. comptipennis* and *P. insularis*) (Konishi 1990, Turrisi and Watanabe 2011). Males of *Pristaulacus emarginaticeps* can be distinguished from these three species by the shape of the subgenital plate, paramere, digitus and penis valve (Fig. 5a-d). Thus, morphology of male genitalia may also be used as a diagnostic character, but further observations of the male genitalia of other species are needed.

## Supplementary Material

XML Treatment for Pristaulacus emarginaticeps
